# Protease activated receptor 2 as a novel druggable target for the treatment of metabolic dysfunction-associated fatty liver disease and cancer

**DOI:** 10.3389/fimmu.2024.1397441

**Published:** 2024-10-11

**Authors:** Gianmarco Villano, Patrizia Pontisso

**Affiliations:** ^1^ Department of Surgical, Oncological and Gastroenterological Sciences, University of Padova, Padova, Italy; ^2^ Department of Medicine, University of Padova, Padova, Italy

**Keywords:** PAR2, proteases, metabolic dysfunction, fatty liver disease, PAR2 inhibition

## Abstract

Metabolic dysfunction-associated fatty liver disease (MAFLD) is spreading worldwide, largely due to unhealthy lifestyles that contribute to the rise in diabetes, metabolic syndrome, and obesity. In this situation, the progression of injury to metabolic steatohepatitis can evolve to cirrhosis and, eventually, to hepatocellular carcinoma (HCC). It is well known that serine protease enzymes with different functions in cellular homeostasis act as signaling molecules that regulate liver inflammation by activating the protease-activated receptors (PARs) family members, expressed on the cellular plasma membrane. Among them, PAR2 plays a central role in the activation of signaling pathways in response to changes in the extracellular microenvironment. Experimental data have provided evidence that PAR2 is involved not only in inflammatory response but also in insulin resistance, lipid metabolism, and cancer. The major aims of this narrative review are addressed to assess PAR2 involvement in inflammation, metabolism, and liver disease progression and to explore possible therapeutic strategies, based on PAR2 inhibition, in order to prevent its biological effects in the context of MAFLD and cancer.

## Introduction

In the recent years, metabolic dysfunction-associated fatty liver disease (MAFLD) has emerged as a major cause of hepatic diseases in Western countries, and it is expected to become the main etiologic factor of liver-related mortality within a few years worldwide (1). Accumulation of triglycerides and free fatty acids within the liver is the hallmark of this pathologic condition, strongly associated with insulin resistance, deregulation of hepatic lipid metabolism, obesity, and metabolic syndrome (2). Lipid accumulation induces liver inflammation through the activation of endogenous signaling pathways and the release of a large number of mediators, including growth factors, chemokines, pro-inflammatory cytokines, and plasma proteins. In this scenario, hepatic macrophages and myofibroblasts cooperate in a finely regulated manner and play a prominent role in sustaining the progression of injury to metabolic dysfunction-associated steatohepatitis (MASH). Approximately 25% of the patients with MASH can evolve to cirrhosis and, eventually, develop hepatocellular carcinoma (HCC) (3, 4). A growing number of observational clinical studies demonstrate that liver cancer sometimes develops in patients with MAFLD, even in the absence of cirrhosis, indicating that obesity and hepatic steatosis are also associated with an increased risk of cancer that can occur also in extrahepatic tissues, including other parts of the gastrointestinal tract itself (5).

Despite promising indications from preclinical studies (6), at present, the only drug recently approved for MAFLD is the selective thyroid hormone receptor-β agonist resmetirom (MGL-3196) (7).

It is well known that serine protease enzymes with different functions in cellular homeostasis, capable of recognizing and degrading extracellular proteins, also act as signaling molecules that regulate liver inflammation by activating the protease-activated receptors (PARs) family members, expressed on the cellular plasma membrane (8, 9).

Computer models have described that PARs, as members of the G-protein-coupled receptor family, have a single chain with seven transmembrane domains connected by three intracellular loops and three extracellular loops. The structure is completed by an extracellular N-terminal domain and an intracellular C-terminal domain. Rather then being stimulated through ligand receptor occupancy, they are activated by the proteolytic cleavage of an N-terminal sequence by several proteases, generating a novel unmasked tethered ligand (TL), which subsequently folds back on the cut receptor molecule, determining its activation. The signal, by a conformational change, spreads via intramolecular binding and activates the signaling cascade response (10, 11). The four members of the PAR family are activated by proteases, among which trypsin and trypsin-like proteases activate only PAR2 (12).

The coupling with proteolytic cleavage of PAR2 by many proteases, such as trypsin and tryptase, is performed at a “classical site” (Arg^36^–Ser^37^), but different proteases can cut into different sites. Therefore, different TL sequences are produced and activate specific signaling pathways which vary from tissue to tissue, a situation known as “functional selectivity” (13–15).

**Figure 1:**
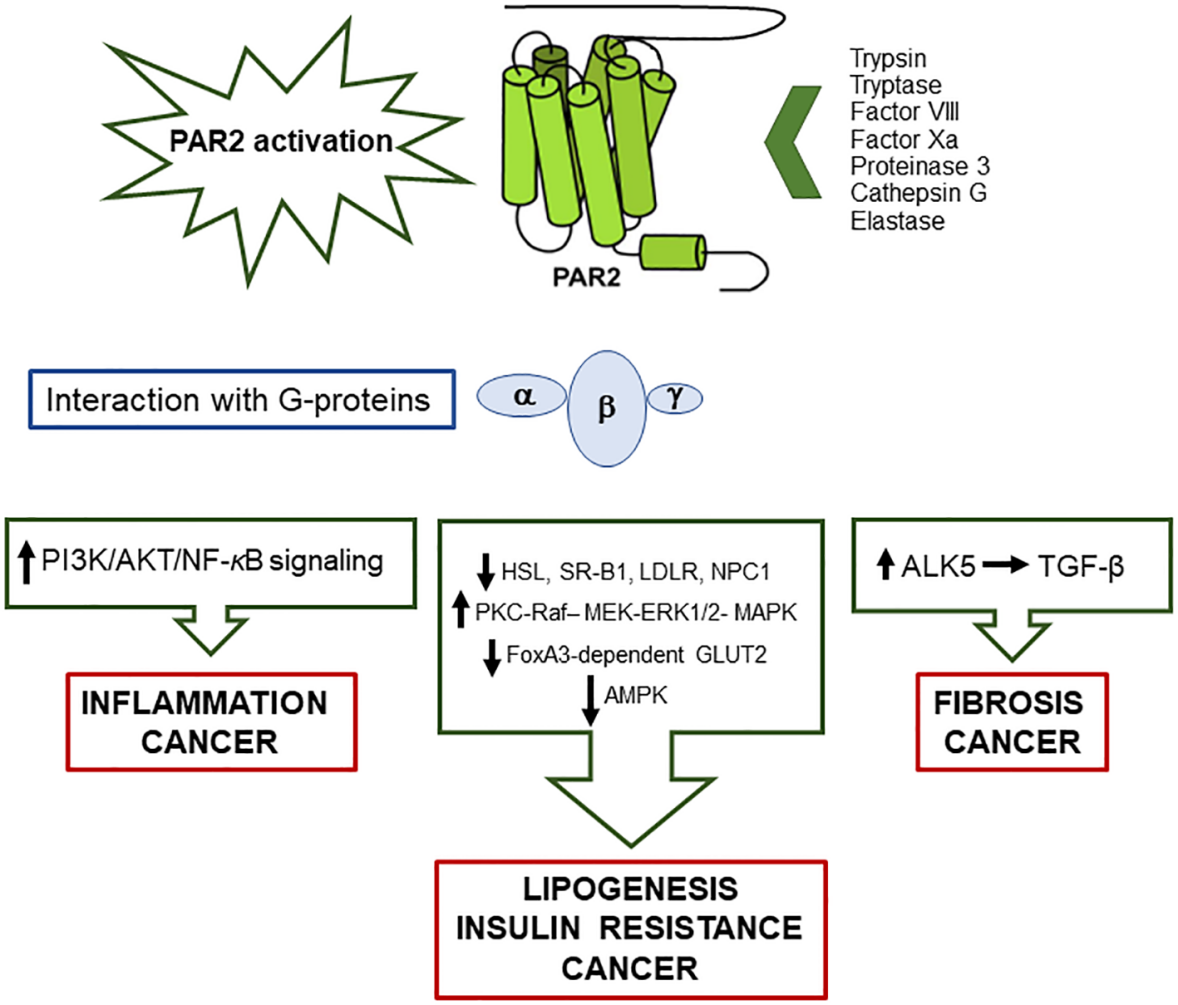
Schematic representation of the main pathways activated by PAR2/G-proteins’ interaction, following protease activation, and involved in metabolic liver disease, insulin resistance and cancer.

Among PAR family members, PAR2 is expressed on macrophages’ surface and exerts a pivotal role in inflammatory processes (9). Mostly, *in vitro* studies have reported that the classical activation of PAR2, through the proteolytic cleavage of the N-terminal domain, unmasks the tethered ligand which triggers mitogen-activated protein kinase (MAPK) activation, with subsequent activation of NFκB via G protein α-subunits’ interaction (16) (**Figure 1**). The activation process can also take place by a G protein-independent pathway via the multifunctional cytosolic β-arrestins, acting as both scaffolding and signaling proteins. β-arrestin-1/2 are transducers that promote the MAPkinase signaling cascades, such as the extracellular signaling kinases 1 and 2 (ERK) and c-Jun N-terminal kinase 3 (JNK3). Once recruited and phosphorylated, they regulate PAR2 activity, interacting with PAR2 intracellular loops and the cytoplasmic tail (C-tail) (17). They contribute therefore to the entity and duration of PAR2-induced cellular responses, mediating the process of the receptor internalization through endocytosis mechanisms (18) and subsequently ubiquitination (19). Phosphorylated PAR2, then uncoupled from the G protein complex, is carried by endosomal vesicles toward the internalization machinery where the receptor degradation occurs. The internalized PAR2 is not recycled, and a newly generated PAR2 is required for quick externalization and activation on the membrane. This process makes the cells not responsive to proteases for a certain period of time, at least until the cell membrane has recharged with newly synthesized and activated receptors (17, 20).

Neutrophil proteinases (elastase, proteinase-3, and cathepsin-G) can selectively modulate PAR2 signaling, cutting downstream of the tethered ligand at distinct cleavage sites: at Ser^68^–Val^69^ for elastase, at Val^62^–Asp^63^ for proteinase-3, and at Phe^65^–Ser^66^ for cathepsin-G. The removal of the tethered ligand disarms PAR2 which, in this way, is no longer available to the subsequent activation of trypsin (21). The complex inflammatory action in response to injury involves neutrophil elastase, but not proteinase-3 nor cathepsin-G, selectively activating the MAPKinase pathway via G_α12/13_, without involving either receptor internalization or the recruitment of β-arrestins (22).

It is worth to note that the lysosomal cysteine protease cathepsin S, present in macrophages, microglial cells, B lymphocytes, and dendritic cells, is regarded as a biased agonist of PAR2 that induces inflammation and pain. Cathepsin S is indeed able to cleave PAR2 at Glu^56^–Thr^57^ distinct site, removing the canonical tethered ligand, disarming PAR2, and preventing trypsin activation (23). In addition, the cleavage exposes a domain of PAR2 with the activation of transient receptor potential vanilloid 4 (TRPV4) and causes hyperexcitability of nociceptive neurons, which induce neurogenic inflammation and pain. Through its action, it promotes PAR2 coupling to G_αs_ and cAMP accumulation, while it has no effect on Ca^2+^ induction, activation of ERK1/2, β-arrestin recruitment, and PAR2 endocytosis (24).

The complex communication network between proteases and their ability to modulate the response by selective cleavages underlines the importance of PAR2 as a central sensor for downstream divergent signaling pathways, and this receptor is advocated as a target for possible therapeutic treatments.

Observational human studies have demonstrated that PAR2 is associated not only with the development of inflammatory diseases (9, 25) but also of insulin resistance (26), lipid metabolism (27, 28). and cancer (29, 30) (**Figure 1**).

This review provides an overview of PAR2 focusing on its role in inflammation, metabolism, lipid accumulation and neoplastic transformation, supporting possible therapeutic strategies aimed to inhibit these biological effects in the context of MAFLD and cancer.

## PAR2 in inflammation and fibrosis

PAR2 is expressed in the majority of immune cells, including both the innate and the adaptive immune system, and is involved in several inflammatory-related conditions (31). As member of G-protein-coupled proteases activated receptors family, PAR2 drives many signal pathways determining acute and chronic inflammatory cascades. Its expression is regulated by various inflammatory stimuli, produced as a defense response in conditions such as autoimmune diseases, neurodegenerative, cardiovascular and respiratory diseases, allergic reactions, sepsis, as well as cancer cell invasion and metastasis (32, 33).

The fact that PAR2 activates the NFκB pathway and drives the NLRP3 inflammasome activation, followed by secretion of proinflammatory cytokines, underlines its importance in the inflammatory response (34, 35).

Most animal studies have shown that PAR2 induces the secretion and release of inflammatory cytokines and tissue mediators, leading to kidney damage (36–38). The study of Sui and colleagues indicates that, in a rat model, PAR2 was able to activate PI3K/AKT/NF-*κ*B signaling, leading to massive inflammatory cell infiltration in the renal interstitium during hyperuricemia-induced renal injury, while PAR2 inhibition provided a protective effect (39).

PAR2 plays a significant role in vascular inflammation and tissue injury, where it is upregulated by inflammatory cytokines, such as tumor necrosis factor alpha (TNFα), leading to endothelium-dependent relaxation in the blood vessels of mice and in the arteries of rats (40–42). PAR2 is indeed expressed in smooth muscle and endothelial cells, where it not only mediates direct contraction and nitric oxide-mediated endothelium-dependent relaxation but also induces smooth muscle contraction of endothelium-denuded preparations of mouse mesenteric arterioles (43).

Using *in vitro* and *in vivo* models and patient samples, Ferrell and colleagues have demonstrated the central role of PAR2, unique among the PARs family members, in the development of inflammatory arthritis (44). An increased expression of this receptor was detected indeed in osteoarthritis and rheumatoid arthritis synovial tissues (45). In line with these findings, PAR2-deficient mice were protected from arthritis for up to a year (46, 47). The overexpression of PAR2 in the synovium and surrounding periarticular tissues was associated to worsening of osteoarthritis, where PAR2 promoted the release of pro-inflammatory cytokines and matrix metalloproteinases (MMPs), resulting in tissue damage, swelling, granulocyte infiltration, and osteophyte formation (48, 49). Supporting the role of PAR2 in rheumatic disease, the increased expression of PAR2 in the monocyte infiltrate was correlated with synovial thickness, edema, and increased levels of IL-6 in synovial explant tissue from patients with rheumatoid arthritis (50).

On the other hand, regarding the respiratory system, PAR2 seems to have a dual role, both pro- and anti-inflammatory. These opposite effects are likely due to the different types of proteases cleaving PAR2 and the different sites of expression of the receptor in the airways (51, 52). Indeed PAR2 has been detected both in bronchial epithelial and in smooth muscle cells, where it upregulates inflammatory signaling and the progression of fibrosis in lung diseases (53). Conversely, its activation may be associated with bronchial protection and bronchodilation through the generation of the anti-inflammatory prostaglandin E2 (54).

The inflammatory processes and fibrogenic events driven by PAR2 have also been observed in the pancreas, where PAR2 mediates pancreatic stellate cells proliferation and collagen production in experimental models (55). In addition, PAR2 participates in the regeneration of endocrine pancreas, leading to islet cell transdifferentiation in the absence of β cells (56). These results are in line with the fact that, in a model of autoimmune diabetes, its presence in β cells was protective against β-cell destruction; however, PAR2 expression within the immune system determined the exacerbation of the disease (57). PAR2 is also upregulated in mouse models of atopic dermatitis, where high levels of tryptase were observed (58). The presence on the skin of exogenous proteases can activate surface PARs, determining protective barrier dysfunction and increased permeability of the epidermis (59). Indeed, in patients with atopic dermatitis, PAR1 and PAR2 were activated in skin keratinocytes, with subsequent triggering of NFκB pathway and release of IL-8, IL-6, and granulocyte macrophage colony-stimulating factor (GM-CSF) (60, 61).

To better understand the function of PAR2, genetically modified animal models were frequently used; however, conflicting results were achieved, depending on the different experimental designs, resulting sometimes in the worsening of the injury and at other times in its improvement. PAR2-deficient mice were indeed protected from liver inflammation in a model of immune-mediated hepatitis (62), while more extensive liver damage was observed following a direct necrotic injury (63). The reciprocal bone marrow replacement showed an upside-down situation, with the PAR2-deficient mice behaving like the wild-type mice and *vice versa*. This seemingly contradictory behavior can be explained by the fact that PAR2 is a sorting point between inflammation and regeneration regardless of gender: when it is activated by an inflammatory stimulus, it determines worsening of the inflammation, while if it is activated by a direct tissue damage it promotes regeneration (64, 65).

## PAR2 in metabolic-associated liver disease

Lipid homeostasis is maintained through a balance between dietary uptake, *de novo* synthesis, and hepatic excretion into the gut (66). Increased synthesis of hepatic cholesterol and/or suppression of reverse cholesterol transport are associated with dyslipidemia, obesity, insulin resistance, and metabolic syndrome, typical features observed in patients with MAFLD. Fatty liver disease, with the appearance of inflammation, can evolve to MASH, which, in turn, can progress to liver fibrosis, cirrhosis, and HCC (67). One of the central players of this scenario is PAR2, expressed on different cell types, including liver stellate cells, hepatocytes, inflammatory cells, and other mesenchymal cells that regulate the response to tissue injury. This receptor is activated not only by trypsin-like proteases but also by coagulation factors VIIa and Xa, when tissue factor is upregulated, as it occurs in subjects with fibrotic liver disease (68). A crucial role in the development of liver fibrosis is played by stellate cells which, through PAR2 activation, pass from a quiescent state to that of active myofibroblasts (69). Activated stellate cells trigger multiple signal pathways to develop a pro-inflammatory and pro-fibrotic microenvironment through the secretion of cytokines, such as TGF-β, TNF-α, and IL-1β and the recruitment of Kupffer cells that acquire an M1 phenotype (70). The production of TNF-α and IL-1β has also the task to induce the expression of PAR2 in endothelial cells, promoting an increased permeability of the vessel wall and white blood cell recruitment (71, 72). In addition, TNF-α upregulates the expression of IL-8, IL-6, and MCP-1, attracting inflammatory cells which propagate local fibrosis and cause additional liver damage (73). At the same time, stellate cells stimulate collagen production and remodeling of the extracellular matrix, leading to the development of progressive liver fibrosis and cirrhosis (74).

Once activated, PAR2 also determines JNK 1/2 phosphorylation which inhibits insulin signaling, resulting in insulin resistance and subsequent liver steatosis associated with increased gluconeogenesis, through the activation of the lipogenic-regulatory transcription factor SREBP-1c (75). Parallel with the activation of SREBP-1c, the AMPK pathway is inhibited, preventing lipid catabolism and the hydrolysis of triglycerides (**Figure 1**). Accordingly, PAR2-KO mice showed downregulation of liver SREBP-1c and activation of phospho-AMPK (28). In line with these findings, in patients with MAFLD, PAR2 expression was significantly increased in the liver, and its expression was correlated to high levels of plasma cholesterol, a result that was confirmed by experimental data in high-fat-diet-fed mice (28).

On the other hand, PAR2-deficient mice under high-fat-diet conditions showed attenuation of hepatic gene expression of *de novo* cholesterol synthesis enzymes, associated with the upregulation of genes involved in controlling cholesterol influx, such as scavenger receptor class B, member 1 (SR-B1), hormone-sensitive lipase (HSL) which hydrolyzes cholesterol esters to free cholesterol, LDL receptor (LDLR), and NPC intracellular cholesterol transporter 1 (NPC1), crucial for liver internalization of cholesterol. These changes were accompanied by suppression of *de novo* lipogenesis, increased β-oxidation of fatty acids, a marked ketogenic shift, and higher fecal bile acid secretion, confirming that the lack of PAR2 is protective against the development of obesity, MAFLD, and hyperglycemia (28). In addition, PAR2-deficient mice showed a decrease of blood glucose levels and of weight gain and an improvement of glycemic parameters, along with increased glucose uptake and storage. These metabolic data were accompanied at the transcription level with an increase of GLUT2 mRNA and of its promoter FoxA3. The inverse correlation between the expression of PAR2 and GLUT2 was also confirmed in patients with diabetes and MAFLD/MASH (76).

## PAR2 in liver cancer

It is well known that liver cirrhosis is the main risk factor for the development of HCC, and this pathological condition is often associated with a hypercoagulable state (77). Coagulation factor VII (FVII) is produced by liver cells and circulates in the bloodstream in an inactive form. Its binding with procoagulant tissue factor (TF) converts FVII into the activated form, leading to the formation of a complex that triggers the coagulation serine protease cascade. TF/FVII complex activates PAR2, which inhibits AMPK-mediated autophagy, supporting malignant transformation of liver cancer cells *in vitro* and *in vivo* (78). The increased PAR2 expression leads to tumor growth and metastasis by PI3K/AKT signaling activation (**Figure 1**).

In addition to the direct effect on cancer cells, PAR2 signaling also affects stromal cells, including cancer-associated fibroblasts (CAFs) and mast cells. CAFs intervene in the processes of malignant transformation, progression, metastasis, and drug resistance, while mast cells produce PAR-2-activating tryptase and TGF-β, which is one of the major cytokines involved in impaired immune response besides its pro-fibrogenic activity (79) (**Figure 1**).

PAR-2-dependent activation of non-cancerous cells can also promote tumor-related neoangiogenesis since stromal cells secrete angiogenic factors such as tryptase, IL-8, and vascular endothelial growth factor (VEGF), which promote PAR2 activation in endothelial vascular cells, determining mitogenic response via MAPK and ERK1/2 pathways (80) (**Figure 1**). In addition, triggering of PAR2 in HCC cell cultures and in tissue-derived HCC cell lines was able to induce hepatocyte growth factor receptor (Met), which plays a central role in the metastatic process (80).

## PAR2 as therapeutic target

The reported data indicate that PAR2 is implicated in numerous pathological conditions and it is a central player of crucial molecular pathways. For these reasons, it could be considered as an excellent druggable target not only for metabolic and fibrosing diseases but also to fight cancer.

Since PAR2’s discovery in the mid-1990s and especially after the resolution of its crystal structure, several strategies have been developed to antagonize its activation, including the use of non-peptide small molecules, peptides, peptidomimetics, pepducins, and antibodies, although none of them has been approved for clinical use yet (81).

The main challenge in inhibiting PAR2 activity arises from the fact that several proteases are capable of PAR2 activation and have specific binding pokets, leading to different conformational changes. Receptor dimerization or hetero-dimerization (e.g., PAR1-PAR2) and cross-talk with other receptors through transactivation are additional problems that have been faced and that are pushing the research to develop novel strategies, including the design of hetero-bivalent ligands (81).

Regarding PAR2 inhibitors in the field of liver diseases, pepducin technology has been developed.

PAR2 pepducins are lipidated peptides comprising a short peptide, tethered to palmitate as hydrophobic moiety, able to target the receptor/G protein interface via an allosteric mechanism. Pepducin PZ-235 was selected as a full PAR2 antagonist since this compound was effective in the inhibition of liver fibrosis, hepatocellular necrosis, reactive oxygen species production, steatosis, and inflammation in experimental NASH (68). Additional studies in diabetic mice have reported that this compound, by blocking PAR2 signaling, resulted in a significant improvement of glycemic indices and HbA1c levels, restoring GLUT2 and Akt activity (76).

**Figure 2:**
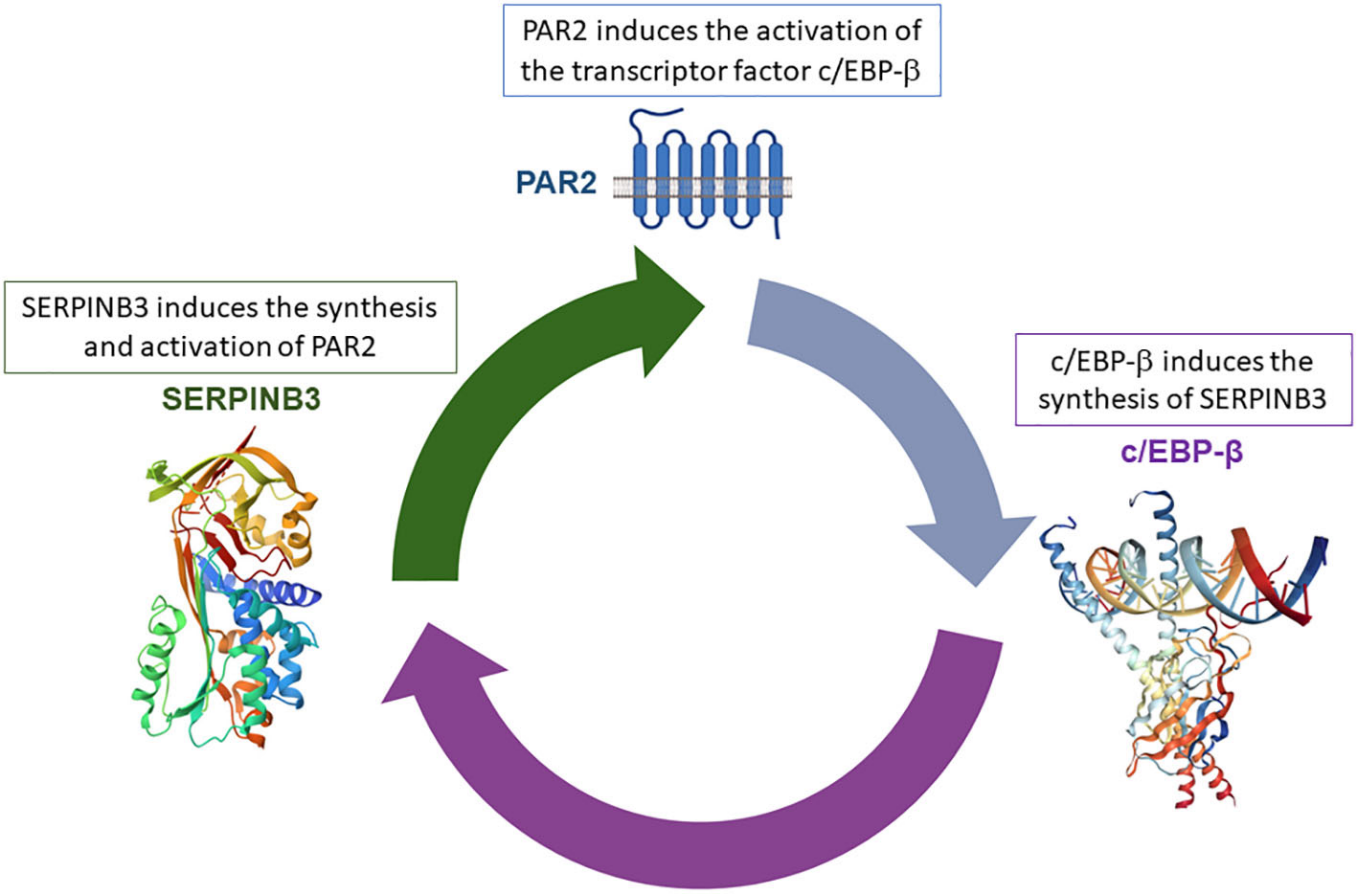
Positive loop between SerpinB3/PAR2 and c/EBP-β. The anti-protease activity of SerpinB3 is essential for PAR2 activation, resulting in increased c/EPB-β transcription factor synthesis, which, in turn, determines the transcription of SerpinB3.

Recently, we have identified a novel PAR2 inhibitor, namely, 1-piperidine propionic acid (1-PPA), which has been shown to be effective against inflammatory processes at very low concentrations. Biophysical and *in silico* analyses demonstrated that 1-PPA binds PAR2 in an allosteric pocket of the receptor inactive conformation, while functional studies revealed an antagonist effect on MAPKs signaling (82). This small molecule was able to reduce lipid accumulation, inflammation, and fibrosis, the hallmarks of MASH progression, by inhibiting c/EBP-β transcription, another pivotal regulator of metabolism, inflammation, and fibrosis (83, 84). This transcription factor induces the synthesis of SerpinB3, a serine protease inhibitor whose antiprotease activity was found essential for PAR2 synthesis and activation (**Figure 2**) (85).

SerpinB3 (also known as SCCA1, squamous cell carcinoma antigen) is a member of the family of serine protease inhibitors (Serpins) which is almost undetectable in normal hepatocytes, while it is upregulated in chronic liver disease (86) and over-expressed in hepatocellular carcinoma (79, 87). In previous studies, SerpinB3 was identified as a critical mediator of liver inflammation and fibrosis since it upregulates the expression of TGF-β1 in chronic liver and lung diseases (88, 89) and exerts a direct pro-fibrogenic action on human liver myofibroblasts in culture by strongly upregulating the expression of pro-fibrogenic genes (including collagen type 1A1, α-smooth muscle actin, TGF-β1, and tissue inhibitor of metalloproteases type 1 or TIMP1) (90).

In addition, studies on transgenic mice, either overexpressing SerpinB3 or carrying a deletion in its antiprotease loop, together with *in vitro* studies on human macrophage cell lines, have shown that SerpinB3 can operate as a pro-inflammatory mediator in two MASH experimental models, induced by feeding mice with methionine/choline-deficient (MCD) or choline-deficient L-amino-acid-defined (CDAA) diets (91).

SerpinB3 expression is upregulated by hypoxic conditions, and its increased transcription is mediated by specific binding of hypoxia-inducible factor (HIF)-2α to the SerpinB3 promoter (92).

Moreover, studies in transgenic mice carrying hepatocyte-specific deletion of HIF2α and analysis performed on MAFLD/MASH patients have shown that HIF2α activation is a key feature of both experimental and human MASH, where HIF-2α levels were strictly associated, in a positive loop manner, with hepatocyte production of SerpinB3, a factor able to deeply affect lipid metabolism (93, 94).

Consistent with previous findings, parenteral administration of 1-PPA in mice fed with MASH-inducing diets determined a remarkable inhibition of PAR2/c/EBP-β signaling, associated with SerpinB3 downregulation. The block of the positive loop between these three molecules determined a marked anti-lipogenic effect, associated with a significant reduction of liver inflammation and fibrosis (85).

## Conclusions

On the basis of the multiple effects of PAR2 in pathological conditions and the preliminary results obtained with PAR2 antagonists, the development of pathway- and receptor-selective PAR2 modulators may be an achieveble goal for the realization of effective drugs that can be useful for PAR2-mediated diseases.
